# FAIM-L - SIVA-1: Two Modulators of XIAP in Non-Apoptotic Caspase Function

**DOI:** 10.3389/fcell.2021.826037

**Published:** 2022-01-10

**Authors:** Elena Coccia, Montse Solé, Joan X Comella

**Affiliations:** ^1^ Cell Signaling and Apoptosis Group, Vall d’Hebron Institute of Research (VHIR), Barcelona, Spain; ^2^ Centro de Investigación Biomédica en Red Sobre Enfermedades Neurodegenerativas (CIBERNED), ISCIII, Madrid, Spain; ^3^ Departament de Bioquímica I Biologia Molecular, Facultat de Medicina, Universitat Autònoma de Barcelona (UAB), Bellaterra, Spain

**Keywords:** synaptic plasticity (LTP/LTD), pruning, XIAP antagonist, axon remodeling, Alzheimer's disease

## Abstract

Apoptosis is crucial for the correct development of the nervous system. In adulthood, the same protein machinery involved in programmed cell death can control neuronal adaptiveness through modulation of synaptic pruning and synaptic plasticity processes. Caspases are the main executioners in these molecular pathways, and their strict regulation is essential to perform neuronal remodeling preserving cell survival. FAIM-L and SIVA-1 are regulators of caspase activation. In this review we will focus on FAIM-L and SIVA-1 as two functional antagonists that modulate non-apoptotic caspase activity in neurons. Their participation in long-term depression and neurite pruning will be described in base of the latest studies performed. In addition, the association of FAIM-L non-apoptotic functions with the neurodegeneration process will be reviewed.

## Introduction

Neuronal apoptotic pathways play crucial roles during the nervous system development (Burek and Oppenheim, 1996) as selective elimination of those neurons that are unable to correctly innervate their targets, ensuring the survival of complete and functional circuits only. We have an extensive knowledge of the pathways that lead to apoptosis. Caspases are the main executioners of apoptosis, and for years their role orchestrating programmed cell death has been broadly reported in physiology and pathology of the nervous system (Lossi et al., 2018; Hollville et al., 2019; Voss and Strasser, 2020).

During the last 2 decades, however, accumulating evidence has been supporting a non-apoptotic and non-inflammatory function of caspases in neurons, essential in processes that occur during the whole life of the organism, as dendritic pruning and synaptic plasticity. Overall, caspases emerge as decisive in every structural change of the nervous system beyond apoptosis, starting from development, remodeling, and finally degeneration (D’Amelio et al., 2011; Hyman, 2011; Unsain et al., 2013; Mukherjee and Williams, 2017).

In the non-apoptotic processes as pruning it has been shown that while active caspases are found in degenerating axons, the cell bodies remain viable (Williams et al., 2006; Simon et al., 2016). To allow neuronal survival, caspase activation is required to be transient and localized to specific cell compartments, such as neurites during pruning upon nerve growth factor (NGF) deprivation or nerve injury (Simon et al., 2012; Unsain et al., 2013; Yang et al., 2013), or synapses during plasticity processes (Hollville and Deshmukh, 2018). Different levels of control can limit caspase to sublethal activation conditions, such as specific and a rapid turnover of proteins that modulate caspase activation (Jiao and Li, 2011; Simon et al., 2016).

Here we will review FAIM-L and SIVA-1, two proteins involved in the complex molecular interplay that regulates caspase activation which our group has shown to be functional antagonists on caspase-activation and consequent functions in neurons.

### FAIM-L and SIVA-1 as Components of the Classic Apoptotic Pathway

The first Fas apoptotic Inhibitory Molecule gene product, FAIM-short (FAIM-S), was identified as a death receptor (DR) inhibitor in immune cells (Schneider et al., 1999). Few years later, a neuronal-specific isoform, FAIM-long (FAIM-L) was described (Zhong et al., 2001). FAIM-S is ubiquitously expressed and is capable of inducing resistance to Fas-mediated cell death in several cell types (Schneider et al., 1999; Sole et al., 2004; Kaku and Rothstein, 2010). Remarkably, in neurons, FAIM-S does not directly protect from DR-induced apoptosis, but it has a role in promoting NGF-dependent neuronal differentiation and branching through activation of ERK and NF-κB pathways (Sole et al., 2004). FAIM-L on the other hand, is expressed exclusively in neurons and differs from FAIM-S by the inclusion of the neuronal exon 2b, which codifies for 22 aa located at the N-Term of the protein (Segura et al., 2007; Coccia et al., 2017). Our group has focused on elucidating FAIM-L mechanism of action, and over the years we have been able to describe part of its relevance in neurons.

FAIM-L blocks DR-induced cell death by two main mechanisms: direct binding to non-stimulated Fas receptor, therefore impairing caspase-8 recruitment to the DISC complex (Segura et al., 2007), and acting on the regulation of XIAP degradation, the main endogenous inhibitor of effector caspases. FAIM-L can directly bind to XIAP’s BIR2 domain and impair its auto-ubiquitination and consequent degradation by the proteasome. Therefore, FAIM-L maintains XIAP levels, enabling it to inhibit effector caspases and to promote survival (Moubarak et al., 2013).

As part of FAIM-L characterization we were able to identify SIVA-1 as an interacting partner (Coccia et al., 2020). SIVA-1 was first described in 1997 in immune cells as an adaptor protein that binds to the cytoplasmic tail of CD27 receptor (Prasad et al., 1997), and has been later found to also interact and modulate signal transduction of different TNFR family receptors (Spinicelli et al., 2002). SIVA-1 has been described to induce extensive apoptosis through multiple mechanisms (Cao et al., 2001; Py et al., 2004, 2007; Zins et al., 2014). Several typical markers have been reported in SIVA-1 cell death induction, such as BAK/BAX translocation to mitochondrial membrane, cytochrome-c release and caspase-3 activation (Xue et al., 2002; Jacobs et al., 2007; Resch et al., 2009). Moreover, SIVA-1 is an inhibitor and interacting partner of XIAP (Resch et al., 2009; Coccia et al., 2020). In our recent work we demonstrated that SIVA-1 induces neuronal cell death through caspase-3 activation, and that it displaces the FAIM-L/XIAP interaction, which can be sufficient to promote XIAP degradation (Coccia et al., 2020).

### FAIM-L and SIVA-1 in Non-Apoptotic Roles of Caspases

Many forms of learning and memory require experience-dependent synaptic adjustments in the hippocampus (Bateup and Sabatini, 2010). The best characterized forms of long-term plasticity of excitatory synapses in response to glutamate signaling are long-term potentiation (LTP) and depression (LTD). In LTP the outcome is a gain in AMPA receptor (AMPAR) expression and increase in synaptic strength, while in LTD, synapses experience a decrease in the number of surface AMPA receptors and weakening of synapses (Beattie et al., 2000). Studies have shown a great conservation between apoptotic and LTD induction pathways. During LTD it has been shown the activation of essential players of the apoptotic pathway, mitochondrial engagement, and cytochrome-c release. Caspase-3 is found to be the essential effector of LTD, which, in fact, is abolished in the hippocampus of caspase-3 deficient mice (Li et al., 2010).

**FIGURE 1:**
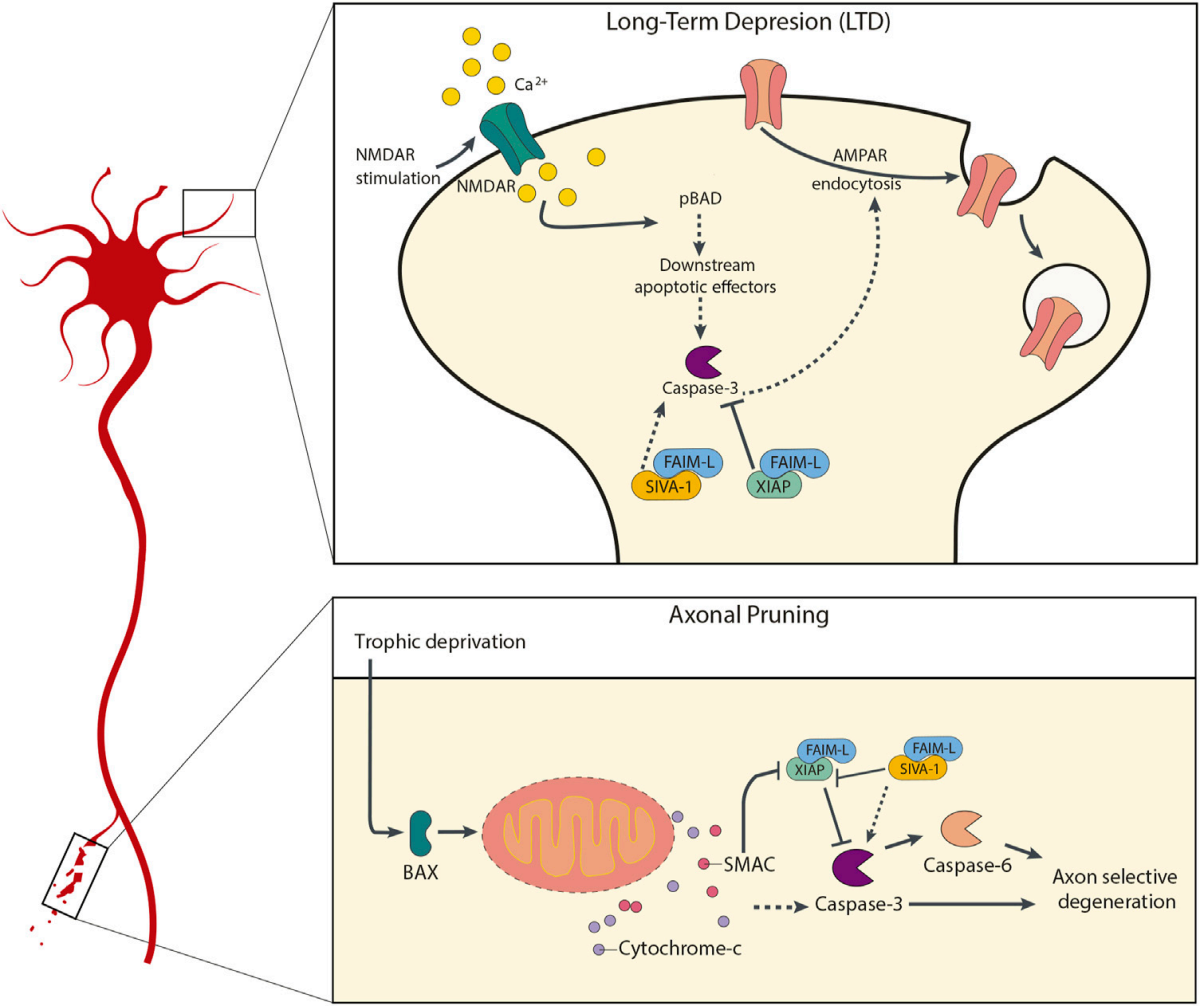
Participation of FAIM-L and SIVA-1 in neuronal plasticity processes. The upper panel diagram represents the molecular mechanism by which FAIM-L and SIVA-1 are involved in AMPA receptors internalization during LTD processes in synaptic plasticity, in response to NMDA stimulation. The maintenance of XIAP-FAIM-L interaction would allow XIAP-mediated caspase-3 inhibition, and therefore prevent the caspase-3-dependent AMPA receptors internalization; a displacement of this interaction mediated by SIVA-1 binding to FAIM-L would allow the activation of caspase-3 and its consequent LTD induction and synaptic weakening. The lower panel diagram shows the similar role of FAIM-L and SIVA-1 in axonal pruning, as in trophic factors deprivation. In this case, an upstream activation of the mitochondrial apoptotic machinery would converge in the activation of caspase-3, whose activity would be regulated by the balance between SIVA-1-FAIM-L or XIAP-FAIM-L binding.

During recent years several modulators of apoptosis have been implicated in LTD regulation. Our group has shown that both FAIM-L and SIVA-1 are able to play a role in AMPAR internalization, but with opposite roles (Figure 1). In our studies we used the extensively described chemical LTD (chLTD) (Li et al., 2004), in which primary neurons undergo LTD changes after cells are trated with NMDA for a short treatment (15min). That is, with NMDA stimulation, chLTD is induced. A rapid increase in calcium levels is triggered by NMDA receptor activation, followed by calcineurin activation. Calcineurin dephosphorylates AMPAR and pBAD, which in turn leads to activation of apoptotic-machinery components and finally caspase-3. The activation of caspase-3 culminates in AMPAR internalization and therefore synaptic weakening (Lee et al., 1998, 2002; Li et al., 2010; Jiao and Li, 2011). In 2016 we demonstrated that FAIM-L overexpression totally abrogates AMPAR internalization in LTD through stabilization of XIAP and consequent caspase-3 inhibition (Martínez-Mármol et al., 2016).

SIVA-1 on the other hand has the opposite effect, as its overexpression is sufficient to decrease GluA2 levels, a component of AMPAR, in a caspase-dependent manner. FAIM-L restores AMPAR levels changes induced by SIVA-1 in hippocampal neurons, indicating that both proteins act on the same pathway.

Another non-apoptotic mechanism in which apoptotic proteins are essential is neurite pruning, essential to sculpt neuronal connections, as it removes excessive or inaccurate projections without resulting in the death of the cell. The mechanism can involve small-scale pruning of axon terminals, or large-scale removal of branches (Luo and O’Leary, 2005).

Pruning is characterized by cytoskeletal destabilization, microtubules disassemble, neurofilament fragmentation and degradation of axonal components. The portion of the axon destined for removal is finally engulfed and digested by surrounding glia (Saxena and Caroni, 2007).

During development of the peripheral nervous system, depriving of NGF only distal axons of neurons promotes axon pruning and remodeling without causing neuronal death (Nikolaev et al., 2009; Cusack et al., 2013). *In vitro* culture of peripheral sensory neurons is a convenient system to study this process, and has been widely used as a model for developmental disease (Campenot, 1977).

Experimental results show that in response to local NGF withdrawal, a pathway that converges with apoptotic initiation is activated. BAX engagement is required to induce cytochrome-c release from mitochondria (Nikolaev et al., 2009), and a caspase-9 to caspase-3 cascade is activated and crucial for apoptosis (Luo and O’Leary, 2005; Buss et al., 2006). Caspase-3, the main effector, activates caspase-6, which plays a significant but subsidiary downstream role (Simon et al., 2012). Caspases finally orchestrate cytoskeleton fragmentation and organelle degradation.

Several mechanisms fine-tune the activation intensity of caspase-3, thereby favoring a transient activity in LTD and pruning, instead of a persistent activation that would commit cells to induce apoptosis. Proteins that participate in apoptotic modulation are found in dendrites and synaptic terminals, which are also equipped with the tools for a rapid and strictly regulated protein turnover, such as proteasome for degradation, translation machinery, and local mRNA (Williams et al., 2006; Ertürk et al., 2014). Mitochondria are essential for activating the pathway that leads to caspase engagement in non-apoptotic activities. These organelles can be found in dendrites, and sometimes in individual spines (Li et al., 2004). Moreover, their distribution and motility are regulated by synaptic activity, allowing for a spatial control of the pathway activation (Li et al., 2004).

During plasticity processes it is essential to have a local and rapid turnover of components of the synaptic proteome, composed by ion channels, neurotransmitter receptors, regulators of synaptic function, and adhesion and scaffolding molecules. Synapses are equipped to have rapid and dynamic changes in protein levels necessary for plasticity. Both translation machinery and proteasomal system are found in synapses, so that protein synthesis and degradation occurs rapidly and independently of the cell Soma - that can be located up to hundreds of microns away (Schuman et al., 2006; Richter and Klann, 2007; Tom Dieck et al., 2014). Like other pro-apoptotic proteins, SIVA-1 is involved in this process by maintaining a partial induction of caspase-3 activation. During LTD, pro-apoptotic BAX and BAD have been shown to be sufficient and necessary for mitochondrial factor release. However, as opposed to apoptotic induction, in this situation BAX translocation to the mitochondrial membrane is not observed, therefore resulting in only a mild engagement of mitochondria that could explain the transient activation of the apoptotic cascade (Li et al., 2010; Jiao and Li, 2011). SIVA-1 regulation, on the other hand, is secured by a rapid calcium dependent induction, and its levels are maintained only for a few minutes after the stimulation (Coccia et al., 2020). Synthesis of new proteins encoded by pre-existing mRNAs in the synapses occurs in response to stimulation, and even if it is not necessary for initiation of AMPAR endocytosis in hippocampal LTD, it is required for AMPAR trafficking for its maintenance (Huang et al., 2005; Hoeffer and Klann, 2008; Di Prisco et al., 2014).

FAIM-L also recently emerged as a regulator of caspase-dependent non-apoptotic activation during long-term synaptic depression (LTD) and neurite pruning (Martínez-Mármol et al., 2016). As other anti-apoptotic proteins such as XIAP and BCL-2, FAIM-L has a crucial role in temporal and spatial restriction of the physiological activation of caspases to local sites and to suppress it so that it does not spread to the whole cell (Bateup and Sabatini, 2010; Li et al., 2010; Martínez-Mármol et al., 2016). Specifically, IAPs (XIAP protein family) can bind to caspases in a sort of IAP-based “clutch system,” in which active caspases are stalled, ready for quick release upon stimuli, bypassing the need for Apaf-1 and apoptosome formation. Together, low Apaf1 engagement and high FAIM-L levels grant a strict control of XIAP post-cytochrome-c inhibition of caspases which allows to stop the wave of caspase activation before it can reach cell Soma (Potts et al., 2003; Martínez-Mármol et al., 2016).

### FAIM-L and SIVA-1 in Pathologies

Our group has found a correlation between FAIM-L levels and Alzheimer’s disease (AD) progression (Carriba and Comella, 2015; Carriba et al., 2015). AD is the principal neurodegenerative disease, whose main hallmarks are extracellular aggregates of amyloid beta (Aβ), intraneuronal Tau neurofibrillary tangles, and progressive neuronal loss. FAIM-L decrease is induced by Aβ oligomers in culture, and it would imply a higher susceptibility to stress insults to neurons. Moreover, since FAIM-L and SIVA-1 counteract each other’s function, loss of FAIM-L would induce a gain of function of SIVA-1 activity, and overall, a deregulation of the pathways that lead to caspase activation.

Over-activation of caspases would afterwards exacerbate the hallmarks of AD, being first extreme plasticity processes in neurons, and finally neuronal degeneration (Chi et al., 2018; Wang et al., 2020; Baranov et al., 2021). Hundreds of studies can be found on the correlation among caspase activation and AD pathological markers, such as increase in production of Aβ (Unsain and Barker, 2015), microtubule associated protein tau phosphorylation and aggregation (Wu et al., 2010), and as consequence, to deficits in cell survival and plasticity pathways. Mouse models of the disease, which overexpress human amyloid precursor protein (APP), have been extremely useful to detect which pathways are deregulated in the disease. Thanks to these models, it was described that memory loss, beginning early in AD, can be attributed to processes previous to cell death. Aβ oligomers induce a deregulation of LTD, which causes disruption of synaptic plasticity, micro-pruning, and spine loss (Fasulo et al., 2000; Gamblin et al., 2003; Rissman et al., 2004; Zhou et al., 2004; D’Amelio et al., 2011; Oh et al., 2013). Given that both FAIM-L and SIVA-1 are involved in these pathways, any deregulation of their functional balance can be determinant for assessing the progression or a potential treatment of the disease.

Recently, a relationship between FAIM and protein aggregation became evident (Kaku et al., 2020) pointing to a function of FAIM in protein homeostasis. FAIM is able to protect against environmental insults that cause accumulation of cytotoxic and aggregated proteins, such as oxidative stress, which are frequently found in the main neurodegenerative diseases. The protective mechanism in this case is reported to be caspase-independent and based on the ability to bind to ubiquitinated protein aggregates and prevent cell damage. Both FAIM-S and FAIM-L prevent Aβ aggregation *in vitro*. Therefore, FAIM-L decrease in AD could also be pathologically linked to a more rapid, aggressive Aβ aggregation. In a Faim-KO mice model (Huo et al., 2016), we identified the presence of ubiquitinated aggregates throughout the retina, a gliotic activation response in the Müller cells, and pronounced vascular leakage that lead to late-onset photoreceptor cell death (Sirés et al., 2021).

Beyond its role in apoptosis, SIVA-1 also regulates other cellular processes, such as cellular migration (Li et al., 2011; Ma et al., 2017), autophagy (Van Nostrand et al., 2015), and proliferation (Ma et al., 2017; Liu et al., 2020). In cancer studies SIVA-1 has been found to have a role as pro- or anti-malignant factor, depending on cellular context (Vachtenheim et al., 2018).

Studies carried out in ovarian and cervical cancer cells show that SIVA-1 suppresses migration and invasion of cancer cells, and overall prevents metastasis through phosphorylation of stathmin, a microtubule destabilizer (Ma et al., 2017; Liu et al., 2020).

In other cases, instead, SIVA-1 has been reported to have the opposite role. Pro-oncogenic role of SIVA-1 has been linked to a negative feedback loop regulation that can induce degradation of p53 (Wang et al., 2013), and to its involvement in mitochondrial respiratory capacity and energy production. Van Nostrand and others showed in non-small cell lung cancer that SIVA-1 knockdown reduces energy production and results in autophagy, suggesting that SIVA-1 is necessary to facilitate tumorogenesis (Van Nostrand et al., 2015).

In line with the findings of SIVA-1 as a protein of relevance in several cellular processes, Jacobs and others reported an essential role of SIVA-1 during embryonic development, during which a tight regulation of cellular proliferation and differentiation mechanisms is essential. They generated a *Siva-1* KO mice and reported that *Siva-1* deficiency results in mid-gestational embryonic lethality, associated with several developmental abnormalities, developmental delay and defects in neural tube closure (Jacobs et al., 2019).

### Available Mouse Models to Study FAIM

For many FAIM-related functions it is not clear which isoform of FAIM (L or S) is responsible for the observed effects. Currently available *Faim*-KO models lack both forms of the protein, and some inconsistency is found in the reports about *Faim*-KO mice phenotypes. Dr. Lam’s group reported FAIM-S regulation of insulin signaling and first generated a FAIM null mouse in 2009 (Huo et al., 2009). These mice present a spontaneous non-hyperphagic obesity, an increased fatty acid synthesis in the liver, and reduced insulin receptor beta, supporting the involvement of FAIM-S in energy homeostasis and insulin signaling (Huo et al., 2016). The obese phenotype described by Lam’s group was not reproduced in a second *Faim*-KO mice generated by Dr. Rothstein’s group (Kaku and Rothstein, 2020), and in our study with the former model some phenotypic characteristics changed based on the genetic background of the mice strain used. However, our group recently identified a retinal neurodegenerative phenotype in Dr. Huo’s model, compatible with the observations on protein homeostasis reported by Dr. Rothstein (Sirés et al., 2021). Several factors can influence the appearance of phenotypes when analyzing mice models, such as genetic background of mice strain, flanking genes modifications during the generation of the mice, KO generation methodology, or environmental factors. The discrepancy on reports about Faim-KO mice makes therefore difficult for us to appoint any phenotype described to the lack of FAIM-L or FAIM-S. For this reason, it would be of great interest to generate new specific mouse models knockout of each FAIM isoform, and more interestingly of FAIM-L because of its selective expression in neuronal cells and relevance in cell-type specific physiology.

## Conclusion

In conclusion, we have reviewed the role of FAIM-L and SIVA-1 as novel regulatory proteins that modulate the activity of caspase-3, involved in a wide range of remodeling processes that are essential for a correct neuronal function. Given the relevance of this fine-tune regulation, any alteration in regulatory proteins may be a possible cause of pathology, and may constitute therefore, a plausible therapeutic target. A better understanding of the mechanisms involved in synapses remodeling will help the development of new therapeutic strategies for neurodegenerative diseases.
